# Students as ecologists: Strategies for successful mentorship of undergraduate researchers

**DOI:** 10.1002/ece3.5090

**Published:** 2019-03-26

**Authors:** Nathan Emery, Amanda Hund, Romi Burks, Meghan Duffy, Christine Scoffoni, Andrea Swei

**Affiliations:** ^1^ Department of Plant Biology Michigan State University East Lansing Michigan; ^2^ Department of Ecology, Evolution and Behavior University of Minnesota St Paul Minnesota; ^3^ Biology Department Southwestern University Georgetown Texas; ^4^ Ecology and Evolutionary Biology University of Michigan Ann Arbor Michigan; ^5^ Biological Sciences California State University Los Angeles California; ^6^ Department of Biology San Francisco State University San Francisco California

**Keywords:** mentoring, professional development, undergraduate research

## Abstract

Guiding undergraduates through the ecological research process can be incredibly rewarding and present opportunities to break down barriers to inclusion and diversity in scientific disciplines. At the same time, mentoring undergraduate researchers is a complicated process that requires time and flexibility. While many academics receive extensive guidance on how to be successful in research endeavors, we pay much less attention to training in mentorship and working collaboratively with undergraduate students. This paper seeks to provide a framework for successfully collaborating with undergraduates including initial recruitment, development of a contract, fostering student ownership of research projects, and submission of a polished manuscript.

## 1 INTRODUCTION

Involving undergraduates in genuine research experiences has tremendous positive impacts on their education and learning outcomes when done well (Linn, Palmer, Baranger, Gerard, & Stone, 2015; Lopatto, 2007). Research experiences can empower students to conduct independent investigations and exercise critical thinking skills while providing opportunities for building diversity and inclusion in the sciences (Davidson & Lyons, 2018; Haeger, Fresquez, & Marsteller, 2016; Parker, 2018). Mentoring undergraduates through research and publication comes with its own set of challenges (Lunsford et al., 2013) but can have many positive effects on faculty mentors (Adedokun, Dyehouse, Bessenbacher, & Burgess, 2010; Burks & Chumchal, 2009; Hall, Walkington, Shanahan, Ackley, & Stewart, 2018; Hayward, Laursen, & Thiry, 2017; Laursen, Seymour, & Hunter, 2012) and can significantly impact scientific progress across disciplines (Rovnyak & Shields, 2017). Particularly for ecologists, collaborating with undergraduates on research should feel natural, as collaborative research occurs frequently across the discipline (Gorham, 2014; Leimu & Koricheva, 2005). However, while mentoring and working with undergraduates comprises one of the core aspects of being a faculty member (Austin, 2002), academics often receive little to no training in mentoring skills and strategies (Hund et al., 2018). As ecologists with positions at a number of different universities, we sought to identify and describe best practices for collaborating with undergraduates on research projects including recruitment, development of a research question, submission of a publication, and to highlight strategies for success that apply to a variety of situations.

Undergraduate students differ from graduate students and postdoctoral researchers (postdocs) in several ways. First, often new to science, undergraduates likely have limited experience working within the scientific process and may be wholly unfamiliar with academic research and culture (Ovink & Veazey, 2011). Second, they may be juggling many outside demands on their time including classes, work, extracurricular activities, and/or family obligations that prevent them from dedicating significant time to research (Fairchild, 2003). Lastly, while graduate students have committed themselves full‐time to scientific research, undergraduates are likely still figuring out what career they want to pursue. Thus, they may justifiably question whether research will help them in their long‐term goals. These unique characteristics of undergraduate researchers reinforce the importance of being flexible, patient and cognizant of students’ individual needs when developing one's mentoring approach.

Although many diverse ways exist to collaborate with students in ecological research, experiences typically fall into two categories, undergraduate research experiences (UREs) and course‐based undergraduate research experiences (CUREs; Auchincloss et al., 2014). CUREs can be a useful way to involve many students in genuine research experiences. However, given the limited time scale of most CUREs (usually as semester at most), students may have difficulty in participating throughout the entire process from question to publication. Targeted at fewer students, more focused UREs provide opportunities for quality 1‐on‐1 mentoring (Lunsford, Crisp, Dolan, & Wuetherick, 2017) and more in‐depth professional development (Shanahan, Ackley‐Holbrook, Hall, Stewart, & Walkington, 2015; Shellito, Shea, Weissmann, Mueller‐Solger, & Davis, 2001). Involving undergraduates in a research program and guiding them through the scientific process from research question to publication submission often involves a complex path of obstacles and opportunities (Laursen et al., 2012). These bumps in the road might be novel for early career mentors, and this paper seeks to provide some guidance for research mentors interested in advising undergraduates through the entire research process.

The six authors of this paper come from diverse institutions and career stages. All have experience collaborating with undergraduates and are passionate about mentoring young scientists through the ecological research process. We have all been student researchers at one point, and we now navigate the role of research mentor at institutions across the United States. We all agree that successful collaboration with an undergraduate is a partnership between the mentor and the student. This partnership can be described by the following quote:

…good supervision is characterized by trusting relationships where students and supervisors share research interests and supervisors provide advice without undermining students’ ownership of projects, resulting in evolving supportive relationships that foster student growth (Roberts & Seaman, 2018)

## 2 A FRAMEWORK FOR SUCCESSFULLY COLLABORATING WITH UNDERGRADUATES FROM SCIENTIFIC QUESTION TO PUBLICATION

**Figure 1:**
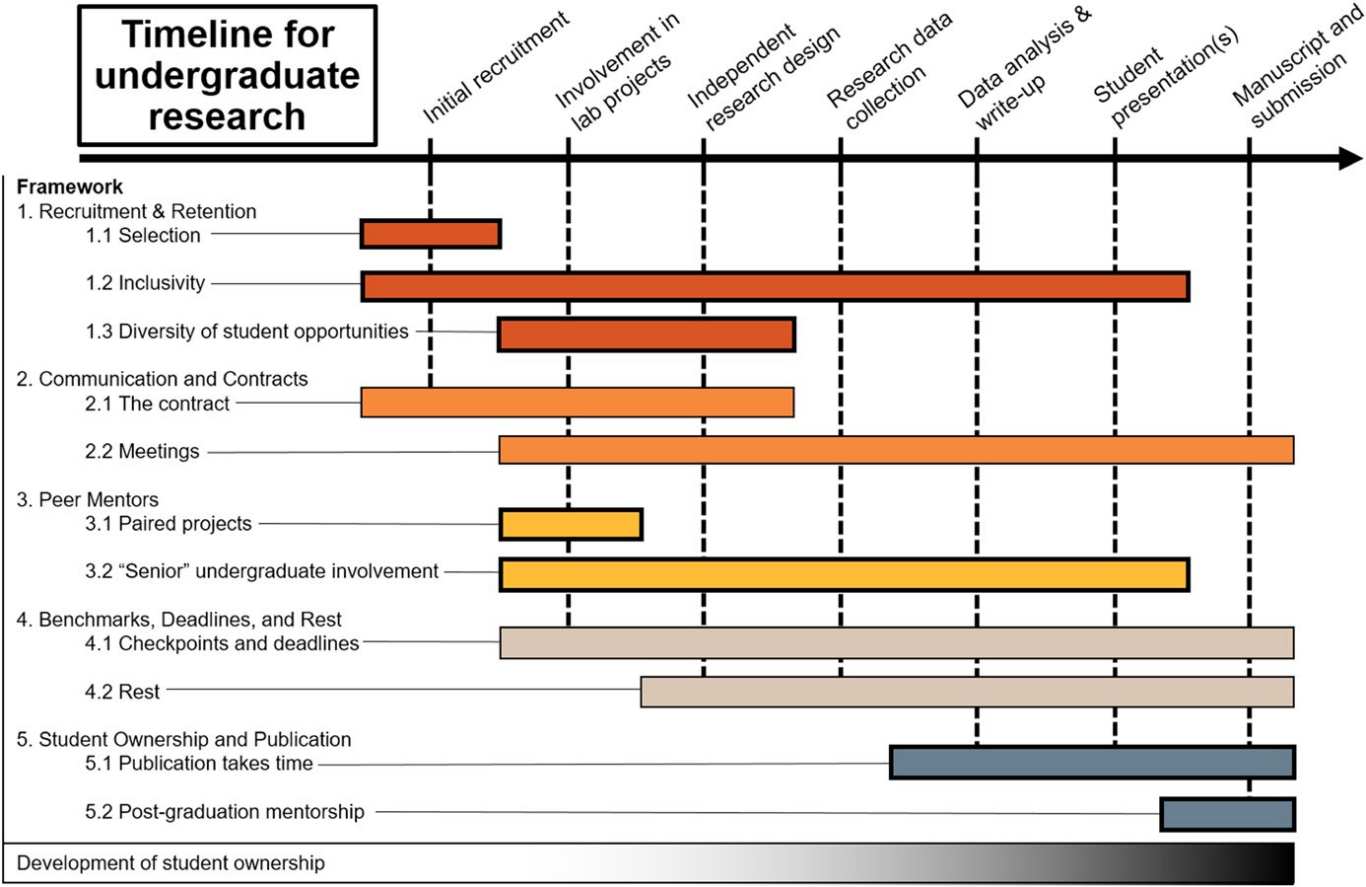
How mentors are involved in the undergraduate research experience over time. Each color represents a different topic described in the text, and the bars indicate when a given mentoring effort generally overlaps with the undergraduate research timeline. The gradient at the bottom represents the development of student ownership over time

We have organized the many facets of collaborating with undergraduates into several sections:

1. *Recruitment and Retention*: When bringing on new students, mentors must take many things to take into account, including their own resources and limitations. Below, we describe strategies and advice on recruiting and retaining students for undergraduate research projects.
2. *Communication and Contracts*: Because clear communication plays such a crucial factor in collaborating successfully with undergraduates, we give specific advice below on how to develop a mutual agreement with a student, and how to maintain effective communication.
3. *Peer mentors*: Involving others in the mentor–mentee relationship can be beneficial for both parties. We discuss how to effectively leverage peer mentors in the process of guiding students through independent research projects.
4. *Benchmarks, Deadlines, and Rest*: The undergraduate research experience, from start to finish, takes a lot of time and varies in its intensity. Mentors offer professional development, boundaries, and rest stops for students as they navigate through the scientific process.
5. *Student ownership and publication*: In addition to student ownership of a project, mentors need to consider how to work with students through the publication process, especially once they have completed their undergraduate degree. We detail ideas and discuss strategies for guiding a project to completion (Figure 1).

### 2.1 Recruitment and retention

Research experience as an undergraduate is an important step for many scientists on their way to graduate school and careers in STEM (Estrada, Eroy‐Reveles, & Matsui, 2018; Hathaway, Nagda, & Gregerman, 2002; Linn et al., 2015). For many young researchers, this experience plays a critical role in building one's identity as a scientist (Lopatto, 2007; Palmer et al., 2015; Robnett, Chemers, & Zurbriggen, 2015). Students arrive to college with different motivations and aspirations and can feel intimidated by scientific research and senior academics. As a mentor, it is incredibly important to build an environment where students feel empowered to ask questions and contribute their ideas (Matthews & Rosa, 2018). Through good mentorship, students gain confidence in their research abilities and develop an identity as a scientist (Davis & Jones, 2017; Linn et al., 2015; Roberts & Seaman, 2018), notably an important component in the retention of underrepresented student groups in science, technology, engineering, and math fields (STEM; Strayhorn, 2012, Wilson et al., 2012, Rainey, Dancy, Mickelson, Stearns, & Moller, 2018). Research experience also yields value to students who do not pursue careers in research, as it gives them first‐hand experience with the process of generating scientific knowledge. While it would be ideal to offer this opportunity to every interested student, quality mentoring requires a considerable amount of time and funding, and resources for projects are finite (Johnson, Behling, Miller, & Vandermaas‐Peeler, 2015). Given these constraints, mentors should think intentionally about how they recruit, choose students for research positions, and offer students opportunities to advance to independent research projects.

#### 2.1.1 Selection

From the mentor's perspective, it seems obvious and beneficial to offer research opportunities to the students who will make the most of the experience, contribute to research, and be a positive member of the research community. However, this selection process can be difficult. In our collective experience, choosing talented, self‐motivated students willing to commit to a long‐term research project increases the likelihood of project completion and publication. Research opportunities should be advertised widely and some students, such as those from groups traditionally underrepresented in STEM fields, may need special encouragement to apply (Crisp, Taggart, & Nora, 2015; Estrada et al., 2016). Advertising in courses and holding open informational meetings where students can learn about different research opportunities in a department provides equal footing for students. When choosing among applicants, mentors need to be aware of biases, both conscious and subconscious, that could discriminate against certain students and influence the hiring process (Hansen et al., 2018; Houser & Lemmons, 2018; Milkman, Akinola, & Chugh, 2015). We recommend that mentors take the time to recognize one's/their own implicit biases through training exercises (See Supporting Information Appendix S1).

Before interviewing potential incoming students, mentors need to take two steps. First, make a list of the different projects students could join/lead in the laboratory and identify any challenges and constraints for each (Table 1). The success of a student in research depends partly on the project assigned: An appropriate project will be one within the student's abilities, while leaving room for them to grow in skills and knowledge (Hunter, Laursen, & Seymour, 2007). Second, as a mentor, identify what student traits hold the most value. Student success is predicted by more than just their current GPA (Dennis, Phinney, & Chuateco, 2005; Komarraju, Karau, & Schmeck, 2009). During the interview, ask the student what motivates her/him/them to do ecological research. We suggest that the best students include those excited about the topic, motivated, hardworking, curious, and reliable. While not an exclusive characteristic of quality students, undergraduate researchers also tend to be more inclined toward pursuing an advanced degree (Lopatto, 2004; Russell, Hancock, & McCullough, 2007). While it might be hard as a mentor to identify these traits in one “interview,” we suggest including other laboratory members in the “interview” process as it may reveal more information and increase the comfort level of the potential student. During the first term of the student working in the laboratory alongside other laboratory members look for signs of motivation, independence, and reliability. The mentor ultimately decides whether the student can oversee an entire project (i.e., become first author on a paper) (Burks & Chumchal, 2009), participate as a co‐author alongside other students that would lead it, help out in the laboratory on lower level tasks, or leave the laboratory to continue different pursuits next term.

**Table 1:** Identifying specific research project challenges and constraints prior to “interviewing” students

Question(s)	Explanation
How many students can you have working together on the project?	This will help you determine how many students you can accept in your lab, as well as accepting students that might need more supervision.
How long will the student need to be in the lab for each day? Will the project require field work on weekends/early mornings/late nights?	Student may need to have an open and accommodating schedule
Can the student work from home? (i.e., computer‐based project)	Students can be more flexible, but need to be very self‐motivated as they won't have the “lab environment” or community to motivate them
How much training will the student require? How difficult are the techniques the student will implement?	If training is intensive and long‐winded, the student may need to stay in your lab for at least one year. Think of how she/he may be able to help train other students on the technique during that year
How many semesters/quarters does the project require the students to be in the lab for?	If the project requires multiple field seasons, the student needs to be able to sign on for multiple years
How time sensitive is the project?	Students will need to be hard‐working and understand the time sensitivity of project goals
How will students access field sites?	Students may need to have a driver's license/be autonomous. Do you have funding to cover travel expenses?

#### 2.1.2 Inclusivity

Mentors must also consider larger issues of inclusivity and diversity when recruiting and hiring undergraduates (Carpi, Ronan, Falconer, & Lents, 2017; Hernandez et al., 2018; O'Donnell, Botelho, Brown, Gonzalez, & Head, 2015). Because research experience serves as such an important step in pursuing a career in STEM, mentors, as the gatekeepers, need to carefully consider their recruitment practices to ensure an equitable and inclusive process. We know that student participation in classrooms can differ across demographics and personalities (Fritschner, 2000), and peer interactions are affected by student characteristics (Eddy, Brownell, Thummaphan, Lan, & Wenderoth, 2015). We can surmise that some students will feel comfortable directly contacting faculty to ask about and pursue research opportunities. By only hiring the most vocal students, we possibly miss out on high‐quality talent and diversity. Good advertising and conscientious hiring practices help diversify undergraduate research but making undergraduate research more inclusive should not stop there.

Too often student research positions go unpaid, or offer very little compensation, particularly over school breaks. This problematic practice limits the students who can participate. For example, students who have less financial support from their families may have to take on jobs to help pay expenses, rather than volunteer for a research position (Fournier & Bond, 2015; Holford, 2017; Shanahan, 2017). The same issues apply to field positions that require students to pay for their own travel or living expenses or have access to a vehicle to get to field sites. We acknowledge that research funding to pay students and cover their expenses may be very difficult to come by; however, this “norm” represents a critical issue that we need to think about and address as ecologists (Fournier & Bond, 2015). As a start, mentors could seek out programs, both at the institutional level and nationally, that focus on promoting inclusion and diversity in undergraduate research and offer funding opportunities. These programs are opportunities to find students, provide financial support, and help build a sense of community among student researchers (see Supporting Information Appendix S2). Funding for undergraduate researchers should also be included in grant budgets and funding agencies need to recognize the importance of this support. Through recruitment practices, mentors have the potential to remove barriers and adopt inclusive practices that chip away at the long‐standing discrepancies in ecology and other scientific disciplines and help retain STEM students (Hurtado, Cabrera, Lin, Arellano, & Espinosa, 2009; Moss‐Racusin, Dovidio, Brescoll, Graham, & Handelsman, 2012).

#### 2.1.3 Diversity of student opportunities

Once students are selected for research positions, their commitment and success depend heavily on how much support they receive from their mentor (Hartmann, Widner, & Carrick, 2013; Hunter et al., 2007; Linn et al., 2015; Russell et al., 2007). Given this, mentors need to consider carefully how many students they can take on and be realistic about their capacity (see Table 1). We believe that fewer, well‐supported students with consistent access to a mentor generally translate into higher quality research, and a better experience for both students and mentors. At the same time, involving multiple undergraduates at once can foster a sense of community and provides opportunities for students to help one other.

Undergraduate research takes on a variety of shapes from a student who helps complete a small task for an existing project to a student conducting an independent research project and authoring a publication. To see a project through to publication, a student and their mentor need to be prepared to commit time and energy over several semesters (if not years) (Morales, Grineski, & Collins, 2017). Scaffolding student experiences to build up to conducting independent research helps ensure the commitment level required to see a project through to completion. New students can join pre‐existing projects working on “low‐stakes” tasks and progressively work up to more “high‐stakes” and independent tasks. During this development period, students require guidance through carefully chosen readings and discussions to better understand how the current research fits into a larger scientific framework. By incorporating new students into the laboratory community (e.g., have them attend laboratory meetings), they also observe more advanced undergraduates and graduate students. Through this process, students learn about the scientific process and gain a more realistic view of what it takes to do independent research. With this knowledge in hand, they become better equipped to decide if an independent project fits into their goals. This process also gives the student time to develop their own scientific interests and preferences. As students develop, mentors can determine which students should move on to independent research and help them to develop a project that provides a good fit.

### 2.2 Communication and contracts

Successful mentoring relationships have a foundation of clear and open communication (Nakamura, Shernoff, & Hooker, 2009). Differences in expectations between the mentor and mentee are the most common factor underlying problematic research experiences (Roberts & Seaman, 2018). When mentors make themselves accessible and students feel comfortable communicating, mentors can better help students through difficult periods, research progress goes more efficiently, and students have more positive experiences. Carrying out scientific research likely differs substantially from a student's past experiences. Most notably, research involves a lot of failure, something that many undergraduate students often fear, work actively to avoid, and typically lack the experience to understand that failure is a teacher (Linn et al., 2015). Clear and frequent communication with a mentor helps students understand that scientific insight often comes from failure (Burger & Starbird, 2012), bolstering student confidence and motivation, increasing satisfaction with their research experience, and fostering a sense of project ownership (Eller, Lev, & Feurer, 2014; Hunter et al., 2007; Linn et al., 2015). One possible way to facilitate a positive attitude about research is by sending an encouraging note to the student from time to time as they persevere through research objectives.

#### 2.2.1 The contract

Communication plays a key part in a successful research experience and many undergraduates may not be familiar with the norms and expectations of how they should interact with their mentors, or may be uncomfortable initiating conversations or meetings. We recommend that mentors take the lead in establishing effective and frequent communication and clearly outlining the research process. While we have developed a base contract based on the collective authors’ experiences, we suggest that mentors develop their own undergraduate mentoring contract that can be shared and discussed with students (see example guide in Supporting Information Appendix S3). The contract clearly outlines expectations, both for the student and the mentor. It should formalize policies on expected behavior, means and frequency of communication, participation in the laboratory community and activities, rules regarding laboratory equipment or resources, required safety trainings, schedule and flexibility of research work, authorship, deadlines, and other important information that will help students succeed and work well with others. Beyond making expectations clear and explicit, discussing this contract early gives the mentor and student a common language and something to refer to when needed. It also helps to keep both the student and the mentor accountable. Most conflicts and issues that arise in mentoring relationships stem from miscommunication and misunderstandings (Burk & Eby, 2010; Eby, Butts, Durley, & Rose Ragins, 2010).

Much of the mentoring contract can be standardized for all students working in the laboratory, but we find the best contracts contain customizable elements for each individual student. Students differ in their personalities, working styles, goals, and backgrounds (Rose, 2005). Student needs will also change as they gain experience and develop as scientists (Thiry & Laursen, 2011). Thus, individual students will need different types of support from their mentor, requiring different mentoring strategies that best fit a given student (Hund et al., 2018; O'Meara, Knudsen, & Jones, 2013; Opengart & Bierema, 2015). Developing a flexible communication plan starts with personalizing components of the mentoring contract and engaging in early discussions with students about the mentoring style that will help them thrive.

#### 2.2.2 Meetings

The initial meetings with a new student provide a critical opportunity for setting the right tone and establishing expectations. Mentors should aim for one‐on‐one weekly meetings with each student, or in some cases, biweekly. Regular meetings provide time not only for tracking progress, taking care of practical research business, and addressing problems early, but also time to build a good mentoring relationship (Baker & Griffin, 2010). During these meetings, mentors should allow space for students to discuss their projects in a broader context, their future goals, general science questions and interests, and questions they have more broadly about academia and research.

Collectively, these conversations contribute to a student's sense of belonging, improve their science communication skills, and help them articulate their goals for science and research (Elgren & Hensel, 2006). Frequent communication and support also improve student mental health and persistence, helping them to persevere when problems arise and research does not go as planned (Estrada et al., 2018; Hernandez et al., 2018). By asking questions and being a good listener, mentors demonstrate to students that their work and ideas have value. Students, in turn, develop ownership over their research with increased incentive to work hard and invest more in their projects (Hanauer, Frederick, Fotinakes, & Strobel, 2012).

Lastly, documenting meetings provides accountability and a record of research progress. One easy way to do this involves creation and maintenance of a shared, online document that highlights the topics discussed at each meeting and sets the short‐ and long‐term goals for the student's research. This can be helpful for the student as a reference and equally useful for busy mentors as it serves to document what previously occurred. If the mentor expects a student to work a certain number of hours, this shared document can also serve as a time and activity log.

### 2.3 Peer mentors

Individual weekly meetings with undergraduates can seem like a daunting time investment, particularly when several students work in the laboratory at the same time. Many senior mentors simply lack time in their schedules to meet with undergraduates as often as they might wish (Baker et al., 2015; Lunsford et al., 2013; Roberts & Seaman, 2018). While understandable, a lack of contact and feedback is problematic as it undermines student confidence and motivation if students perceive a lack of interest, rather than a lack of time. When available, sharing the responsibility of mentoring with a postdoc or graduate student, particularly if they work on similar projects, can increase frequency of feedback and provide professional development for the junior mentors. This allows the undergraduate to still have regular access to a mentor and would also be a chance for the postdoc or graduate student to gain valuable mentoring experience (Dolan & Johnson, 2009). In this situation, faculty mentors should check in regularly to ensure that the student receives the support they need. It is also important that mentors have discussions with their graduate student or postdoc about mentoring best practices and support further mentorship training for these early career scientists (Dooley, Mahon, & Oshiro, 2004; Hund et al., 2018; Weigel, 2015). These training opportunities are sometimes difficult to find, although many institutions provide mentorship resources or mentoring programs. Some training materials are freely accessible online, such as the mentoring manual from Pathways to Science (https://pathwaystoscience.org/manual.aspx) or the *Entering Mentoring* training curriculum developed by the University of Wisconsin, Madison (https://cimerproject.org/#/curricula/training-materials).

Laboratory productivity depends strongly on building a friendly community among students, staff, and faculty and establishing a culture of hard work and scientific ethics. Indeed, the friendlier and more supportive students act toward one another, the more each learns, and the more motivated and hardworking they become (Kobulnicky & Dale, 2016). Peer mentoring has long been studied as a means of helping undergraduates succeed (Budge, 2006; Nicholson et al., 2017) by helping students work through periods of failure or frustration (Baker, Cluett, Ireland, Reading, & Rourke, 2014) and reducing barriers to seeking help (Gross, Iverson, Willett, & Manduca, 2015). If students do not get the chance to know one another, they may become less motivated and not put in the extra mile required in research. Having a socially well‐adjusted laboratory group with fun activities such as potluck dinners, karaoke, or sporting events, for example, also serve as a mentor's future recruitment tool as new students witness the community established in the laboratory. Incorporating students into a community of mentors within a laboratory group and beyond can improve undergraduates’ performance, confidence, and sense of belonging, which play particularly important role in retention of underrepresented minorities and first‐generation students (Good, Halpin, & Halpin, 2000; Kobulnicky & Dale, 2016). Peer mentors typically function in two types of roles, either as a research partner (paired projects) or as a “senior” undergraduate in the laboratory (“senior” researchers).

#### 2.3.1 Paired projects

Pairing students on projects often provides a good way to foster a friendly and supportive laboratory environment and increase research productivity and enthusiasm. Peer mentoring experiences have positive impacts for all students involved, building confidence, motivation, and communication skills (Lopatto, 2010). Collaboration is an essential part of scientific research and is becoming increasingly important in the field of ecology (Goring et al., 2014; Perez & Hogan, 2018). By working as a team, undergraduates have the opportunity to develop and practice the skills necessary for collaboration. Team‐based research, while providing multiple benefits, could come at the cost of independent ownership and development of each student. We suggest a possible compromise is to have each student responsible for different parts of a larger project, specialize on different aspects of the same project, or have them give separate presentations at the end of a term. Even if accomplished in a pair setting, the satisfaction of providing solid contributions could guide the student toward a career in a scientific discipline (Kobulnicky & Dale, 2016; Russell et al., 2007).

#### 2.3.2 “Senior” undergraduate involvement

One way that mentors can recognize and reward the progression of undergraduate researchers as they gain experience and grow as scientists is to give them increasing responsibility and place them in leadership roles (Shanahan et al., 2015). Mentors may assign “senior” undergraduates in the laboratory with a number of tasks that keep the research laboratory functional. Such delegation acts demonstrate the mentor's trust in the student and make it clear that the student is an essential part of the research team, which increases their sense of self‐worth and belonging. For example, as part of their contributions to the laboratory, “senior” undergraduates may conduct routine inventories of supplies, oversee animal care, or perhaps even update a laboratory website. “Senior” undergraduates can also be given the responsibility of training new students in the laboratory. In this case, mentors often treat responsible “senior” undergraduates more along the lines of graduate students, which prepares them well for the transition to graduate school if they choose to pursue a career in STEM. This experience provides the opportunity to practice mentoring and science communication skills, while giving new undergraduates role models (Kobulnicky & Dale, 2016). New students may also feel more comfortable learning from and asking peers for help compared to more senior mentors (Cutright & Evans, 2016; Zaniewski & Reinholz, 2016). Lastly, “Senior” undergraduates play a key role in “laboratory memory” or “institutional history” as long‐term projects continue, but students rotate in and out of the laboratory. Over time, the new students in the laboratory learn what it takes to conduct collaborative research and take on new roles as they in turn become more experienced.

### 2.4 Benchmarks, deadlines, and rest

Deadlines, self‐imposed and otherwise, act as important regulators of time for all researchers, but especially for undergraduate students who are learning time management and often juggle far more activities and responsibilities than mentors may realize. In addition to their coursework, undergraduates may have jobs to help pay for expenses, family responsibilities, school clubs, or other obligations (Berker, Horn, & Carroll, 2003; Fairchild, 2003). Therefore, we find it exceedingly important to set clear and reasonable benchmarks for student research activities (Shanahan et al., 2015), while recognizing that undergraduate research takes time. Limitations on student time and availability probably pose one of the most challenging aspects of working with undergraduate students, but it need not be an insurmountable barrier. Providing short‐term tangible goals, frequent check‐ins, long‐term objectives, and rest stops along their journey all help students progress through their research.

#### 2.4.1 Checkpoints and deadlines

As described earlier, clear communication underlies setting reasonable deadlines that advance the research (Linn et al., 2015; Reed, 2018). Examinations, illnesses, or holiday breaks can often disrupt progress, so we reiterate the importance of meeting regularly with undergraduates to discuss progress, problems, and to adjust expectations and workloads as necessary. Students (as well as faculty) often start out overly optimistic about what they can do with their limited time. When students do not meet deadlines, they may not want to admit their mistake as it makes them feel like they have failed. Frequent meetings can help the student and the mentor realize an unrealistic pace or goal earlier, rather than later, and then adjust. We do not find it unusual to shift or recalculate deadlines and timelines based on these meetings. By doing so, mentors keep the research moving and help their student stay focused and motivated.

Collaborative research with students is a balance of flexibility and clear benchmarks for progress. One example benchmark includes a contractual agreement for an end‐of‐term presentation or write‐up to present to the laboratory or colleagues. Presentations, even when given to a small intimate group, can be immensely helpful in motivating students to accomplish a research objective. Presenting in a laboratory meeting is important for students before they present at larger venues such as off‐campus regional or national scientific meetings. Informal laboratory presentations provide opportunities to assess efforts and progress in a casual atmosphere and, importantly, provide a chance to reward students for their successes, accomplishments, and hard work. After the presentation, the mentor and colleagues should provide truly constructive feedback. No doubt, students will have some difficulties in their research methods or data presentation. The mentor's responsibility includes providing quality feedback without being overly judgmental or critical in expectations that go beyond the experience of the average undergraduate (Estrada et al., 2018). These early presentation experiences help shape students’ confidence and a sense of belonging, both of which contribute to STEM retention of underrepresented groups (Gray, 2000; Perez, Cromley, & Kaplan, 2014; Shanahan et al., 2015).

#### 2.4.2 Rest

Conducting research at any stage can be physically and emotionally draining. Although easier said than done, we acknowledge the importance of providing students (and mentors) a chance to rest. Intervals between academic terms provide obvious opportunities for such breaks. Although it can be tempting to have students work through school breaks when they are free from their coursework responsibilities, many students may greatly benefit from the opportunity to rest and recuperate from their research. Furthermore, as one of the ten salient practices for undergraduate research mentors, Shanahan et al. (2015) argued that mentors need to balance clear and high expectations with emotional support and an appropriate personal stake in the lives of their students. Undergraduate students experience many obstacles during their college experience and mental health issues are common (Hunt & Eisenberg, 2010). Maintaining awareness of your students’ mental health and ensuring rest stops helps provide better balance in their lives as well as the laboratory community. This is not only important for student health, but is an opportunity to establish the expectation of a good work–life balance as the student progresses in their career (Tan‐Wilson & Stamp, 2015).

### 2.5 Student ownership and publication

Undergraduate perceptions of independence and ownership over research projects can increase confidence, retention, and positively influence students’ intentions to pursue a career in research (Corwin et al., 2018; Hanauer et al., 2012; Hernandez et al., 2018). The longer students engage in a research project, the more likely they are to develop feelings of ownership. Roberts and Seaman (2018) identified student ownership as a central theme contributing to a good relationship between research mentor and student. As students gain more responsibility and positive reinforcement from mentors, their sense of ownership should grow (Shanahan et al., 2015).

Managing student ownership undoubtedly comes with its own set of difficulties. As the project progresses, the mentor needs to make thoughtful decisions about the feasibility of guiding a student's project through to publication (Burks & Chumchal, 2009). When the mentor clearly depends on publication of the work for advancement, then the extra time necessary to shepherd a student‐authored work to publication may negatively affect the mentor's motivation (Hardré, Beesley, Miller, & Pace, 2011). In those cases, we recommend that students serve as co‐authors until the mentor establishes more security in her/his/their position. On the other hand, primarily undergraduate institutions often recognize and reward mentors that successfully include undergraduates as co‐authors or mentor students to earn the position of first‐authors. Thus, a multitude of reasons, including their own experience, will drive a mentor's negotiation of authorship and ultimate decision to publish with undergraduates (Burks & Chumchal, 2009).

#### 2.5.1 Publication takes time

While many undergraduate projects never reach the submission phase, undergraduates routinely contribute to peer‐reviewed publications across fields. In the biomedical sciences, Morales et al. (2017) found that several characteristics of mentors and students led to greater productivity in terms of publications: (a) students and mentors worked together for more than a year; (b) mentors found it rewarding to work with students; and (c) mentors possessed more experience in both publishing and higher education. Interestingly, when biomedical faculty mentored black or disabled students, they achieved a significantly higher rate of successful publication (Morales et al., 2017). The authors speculated that a diversity in team performance or a stronger commitment on part of the faculty member or student contributed to this result.

The road to quality peer‐reviewed publication is long and writing with undergraduates often further extends the journey (Burks & Chumchal, 2009). The slow pace of publications can be particularly difficult during the review process. Across the last 40 years, Powell (2016) reported 100 days as a consistent average review time for articles published in PubMed. While this average wait of three and a half months does not seem long for experienced researchers, it feels much different from the undergraduate perspective. This average time to review occupies an entire semester of a typical four‐year undergraduate education and does not take into account time for revisions. Consequently, even in a best case scenario, undergraduates would likely need to submit a paper within the first semester of their last year to see the article in print by the time they graduate. As this submission scenario is unlikely given a student's commitments in their last semester, paper writing and publication can often spill over into postgraduation territory.

#### 2.5.2 Postgraduation mentorship

Working with students after they graduate introduces several new challenges for the student–mentor relationship. These include finding time to meet, tackling complex tasks with less supervision, or working without the logistical support of the institution. Without routine face‐to‐face meetings, the importance of good communication and accountability increases exponentially. Former students often encounter new conflicting demands and face a choice between their new postgraduate obligations and their prior commitments and investments. Mentors too must contend with time dedicated to their current students, while still keeping track of recent graduates. We suggest that mentors and students develop a new plan for communication and work flow postgraduation. Establishing a consistent schedule for communication may prevent procrastination or loss of motivation that can occur postgraduation.

Mentors and students often take on‐campus resources for granted, including access to primary literature, digital storage, and specific software. Before the student graduates, students need to arrange a plan to access electronic library resources, software licenses, and dedicated cloud storage space to back up their research and work on the manuscript. Virtual communication may also be disrupted if the partnership relies on university‐licensed software or email services with expiration dates so it is important to establish a line of communication that works for both the mentor and the college graduate.

## 3 CONCLUSION

As institutional and faculty support for undergraduate research in ecology grows, potential mentors need to be prepared to guide students through the complicated process. Research experiences have numerous benefits to mentors and students alike, including the breaking down of barriers to inclusion and diversity in the sciences (Jones, Barlow, & Villarejo, 2010; Nagda, Gregerman, Lerner, Hippel, & Jonides, 1998). This paper sought to provide a working framework to guide academic mentors as they collaborate with undergraduates from developing a research question to submitting a publication. The future of research lies with the younger generation of scientists. Effective mentorship in research experiences will only improve academia and drive scientific progress.

The publication process more closely resembles a marathon than a sprint, an intimidating concept for many students. Scientific publication as an enterprise, and even more so when including undergraduate researchers, takes drive, persistence, and patience often coupled with a sense of humor (Burks & Chumchal, 2009; Fox, Kuster, & Fox, 2017). As mentors experienced in publishing with undergraduates, we all feel it is worth the effort and hope that the advice in this article makes it a little bit easier. While unpublished science reflects unfinished science and publication is the ultimate goal, not all undergraduates will reach that goal, and the journey they take along the way will be incredibly beneficial for their professional development regardless of publication success.

## CONFLICT OF INTEREST

None declared.

## AUTHOR CONTRIBUTIONS

NE designed, organized, wrote, and edited the manuscript. AH wrote, edited, and contributed supplemental material. RB, MD, CS, and AS wrote and edited the manuscript.

## Data Availability

There are no data associated with this article.
