# Supplementary material for: Students as ecologists: Strategies for successful mentorship of undergraduate researchers
Source: Ecol Evol. 2019 Mar 26;9(8):4316–26. doi: 10.1002/ece3.5090 (PMC6476758; doi:10.1002/ece3.5090)
Supplement: Supplementary file 1 [file ECE3-9-4316-s001.docx]

**Appendix S1. Links with resources for implicit bias training:**

*We found these sources to contain useful academic and general resources, but this is by no means an exhaustive list. Check with your institution for internal resources or opportunities for additional in-person implicit bias training.*

- **National Academy of Science Engineering Medicine:** <http://sites.nationalacademies.org/pga/cwsem/PGA_161607>

- **Berkeley Lab:** <http://diversity.lbl.gov/resources/implicit-bias-awareness/>
- **Boston University School of Public Health:** <https://www.bu.edu/sph/news-events/community-events/diversity-and-inclusion-events/unconscious-bias-workshops/diversity-and-inclusion-resources/>
- **Northwestern:** <https://www.northwestern.edu/provost/faculty-resources/faculty-search-committees/unconscious-bias.html>
- **Brown:**
  <https://www.brown.edu/sheridan/teaching-learning-resources/inclusive-teaching/implicit-bias>
- **University of California, San Francisco:** <https://diversity.ucsf.edu/resources/unconscious-bias-resources>
- **GenPort:** <http://www.genderportal.eu/blog/11-recommended-resources-anti-gender-bias-training>
- **Fair Play:** <https://fairplaygame.org/resources/>
- **University of California Los Angeles**: <https://equity.ucla.edu/know/implicit-bias/>
- **University of Rochester:** <https://www.rochester.edu/college/faculty/resources/bias.html>

**Appendix S2. National programs for undergraduate research**

*Here we list several national programs that support and promote diversity in undergraduate research. Some of these programs will be useful to support students doing research in ecology labs, while others might be useful to recommend more broadly to biology students who are interested in research experience. Many universities and departments have their own programs, so make sure to check what is available locally.*

- **National Science Foundation Research Experiences for Undergraduates (NSF-REU):** <https://www.nsf.gov/crssprgm/reu/>
- **National Institutes of Health Undergraduate Scholarship Program (NIH-UGSP):** <https://www.training.nih.gov/programs/ugsp>
- **National Institutes of Health Summer internship Program in Biomedical Research (NIH-SIP):** <https://www.training.nih.gov/programs/sip>
- **National Institutes of Health Research Initiative for Scientific Enhancement (NIH- RISE):** <https://www.nigms.nih.gov/training/RISE>
- **Big Ten Academic Alliance Summer Research Opportunities Program (B1G-SROP)**: <http://www.btaa.org/resources-for/students/srop/introduction>
- **U.S. Department of Energy Science Undergraduate Laboratory Internships (DOE-SULI):** <https://science.energy.gov/wdts/suli/>

**Appendix S3. Guide to developing your own undergraduate mentoring contract**

As every mentor and student is different, a mentoring contract will be unique and personal. It is important for you to take the time to formalize your own mentoring philosophy and think about how your lab group best functions. Here, we outline suggested topics to think about as you compose a contract. We encourage you to think through these different questions and ask yourself how undergraduates can be successful as part of your research group. What are the “unwritten” rules and expectations that they need to know? We feel that a good mentoring contract should be a living document that can evolve through time, incorporate feedback from lab members, and be adapted to fit the needs of individual students. This document can also serve as a guide in your initial discussions with new undergraduates.

**Introduction and overview:**

- What type of lab environment are you trying to cultivate?
- Why do you think undergraduate research is important?
- What role do undergraduates play in your research program?
- How can having undergraduates work in your lab be valuable to both the students and the lab as a whole?

**What undergraduates can expect from you as their mentor:**

- What are your responsibilities as a mentor?
- What type of feedback can students expect and in what timeframe?
- How will you help students structure goals and timelines for research?
- What are your responsibilities in cases of inappropriate behavior or harassment (ensuring a safe working environment)?
- What are your and the student’s personal strengths/weaknesses that can be addressed throughout the project?

**Types of involvement:**

- What are the different ways that undergraduates are involved in your research program (lab work, fieldwork, class credit, independent research, honors thesis)? How do students access, or earn these different opportunities?

**Communication:**

- How often should undergraduates expect to meet with you (or their secondary mentor)?
- How should undergraduates communicate problems or questions that arise outside of scheduled meetings?
- How much lead time is expected for students to ask for feedback and provide materials?
- What are the expectations regarding email communication (when can students expect a response, how quickly are they expected to respond, should emails be formal or informal)?
- What issues should be discussed over email vs. in person?
- Are there other forms of communication that are okay to use?
- What are the expectations around students talking to lab members outside of scheduled meetings (e.g. should they come talk to you if you are working in your office? When is this okay or not okay?)

**Behavior and research expectations:**

- What are the expectations around scheduled time doing research?
- What should the student do if they are going to be late, or will not make it to scheduled research time or meetings?
- What are the expectations surrounding deadlines and what should the student do if research timelines or deadlines will not be met?
- How will you assess progress on student projects?
- What types of decisions do you expect students to make on their own, vs. decisions that should be checked with you first?
- How should students prepare for one-on-one meetings?
- What are the policies regarding cell phone use, music, food, pets, etc?
- What are the lab guidelines for respect and treatment of others?

**Being part of a research community:**

- How can undergraduates be supportive members of their research community?
- Are students expected to attend other group events (such as weekly lab meetings, or departmental seminars), and if they do attend, are they expected to participate in certain ways?
- Are research projects generally collaborative or do students work on independent projects?
- How can conflict within your lab group be avoided, and if it arises, resolved?

**Policies surrounding equipment, space, and resources:**

- What should students do if equipment they are using is not working, or breaks, especially if they are the one that breaks something (it will happen)?
- Are there areas of the lab that students should avoid, or need to take special precautions in?
- How can students avoid wasting resources, particularly expensive ones?
- How should students work with others using shared spaces or resources?

**Publishing and authorship:**

- How are decisions made regarding authorship and authorship order of research manuscripts?
- Under what circumstances are students included as authors or encouraged to lead manuscripts?

**Letters of recommendation:**

- Explain that writing letters of recommendation is part of your job as a mentor.
- How long should students work with you before requesting a letter of recommendation and what additional information should they provide?
- How much notice should students give before a letter is due?

**Other topics to consider:**

- Expectations for things like funding, conferences, outreach, or fieldwork.
